# Factors associated with gastrointestinal dysmotility in critically ill patients

**DOI:** 10.1515/med-2023-0820

**Published:** 2023-10-05

**Authors:** Nemanja Petrović, Miodrag Žunić, Ana Pejčić, Miloš Milosavljević, Slobodan Janković

**Affiliations:** Department of Pharmacology and Toxicology, Faculty of Medical Sciences, University of Kragujevac, Svetozara Markovića 69, 34000 Kragujevac, Serbia; Department of Clinical Pharmacology, University Clinical Center Kragujevac, 34000 Kragujevac, Serbia; Department of Pharmacology and Toxicology, Faculty of Medicine, University of Maribor, 2000 Maribor, Slovenia; Department of Anaesthesiology, Intensive Care and Pain Management, Maribor University Clinical Center, 2000 Maribor, Slovenia

**Keywords:** gastrointestinal dysmotility, critically ill patients, risk factors, intensive care unit

## Abstract

Critical illness may disrupt nutritional, protective, immune, and endocrine functions of the gastrointestinal tract, leading to a state of gastrointestinal dysmotility. We aimed to identify factors associated with the occurrence of gastrointestinal dysmotility in critically ill patients. A cross-sectional retrospective study was conducted, using patient files as a source of data. The study included 185 critically ill patients treated in the intensive care unit of the University Clinical Center, Kragujevac, Serbia, from January 1, 2016, to January 1, 2022. Significant risk factors associated with some form of gastrointestinal dysmotility were acute kidney injury (with paralytic ileus, nausea, vomiting, and constipation), recent abdominal surgery (with ileus, nausea, vomiting, and constipation), mechanical ventilation (with ileus, and nausea), age (with ileus and constipation), and use of certain medication such as opioids (with ileus, gastro-esophageal reflux, nausea, vomiting, and constipation), antidepressants (with ileus, nausea, and vomiting), and antidiabetics (with ileus). On the other hand, Charlson comorbidity index had divergent effects, depending on the form of gastrointestinal dysmotility: it increased the risk of gastro-esophageal reflux but protected against ileus, nausea, and vomiting. In clonclusion, recognition of factors associated with gastrointestinal dysmotility should initiate preventative measures and, thus, accelerate the recovery of critically ill.

## 1 Introduction

Patients who suffer from, or are at risk of developing life-threatening conditions or diseases, are considered critically ill. As a rule, critically ill patients are treated in intensive care unit (ICU), where the attention of physicians is mainly focused on respiratory and cardiovascular functions [1]. However, critical illness also may disrupt nutritional, protective, immune, and endocrine functions of the gastrointestinal tract (GIT). Diarrhea, nausea, vomiting, gastro-esophageal reflux, abdominal distension, ileus, constipation, and high residual gastric volume, are features of GIT dysfunction classified to the category of gastrointestinal dysmotility (GID). Patients presenting three or more clinical manifestations of GIT dysmotility have a threefold increase in the risk of death [2]. GID is estimated to affect approximately 60% of critically ill patients [3].

Gastroparesis, or slowed gastric emptying occurs with a prevalence of 4–5% in the general population, while among critically ill patients in the ICU, it is encountered more frequently [4]. Acute intestinal pseudo-obstruction, also known as Ogilvy's syndrome, is an acute dilatation of the colon without any mechanical obstruction; it significantly increases mortality [5]. Other modalities of GID are also frequently encountered and may hamper the recovery of critically ill patients. The etiology of gastrointestinal motor dysfunction in critical illness is unclear, but it is likely multifactorial. Factors associated with GID in earlier studies were burns [6], head injuries [7], sepsis, polytrauma [8], chronic diseases like diabetes mellitus type 2 [9], electrolyte abnormalities, advanced age, gender, some medication (such as opioids or alpha-adrenergic agonists), recent abdominal surgery, circulatory shock, and increased plasma levels of some cytokines [10]. However, not all studies confirmed the influence of these factors, and a number of other potentially important ones were not investigated to date.

Considering the significant controversies concerning risk factors and their interaction, our study aimed to test known predictors and investigate some new potential predictors of GID in critically ill patients, as well as to quantify the strength of their effects.

## 2 Methods

A cross-sectional retrospective study design was used for the study. The study population consisted of critically ill patients treated in the ICU of the University Clinical Center Kragujevac in Kragujevac, Serbia, from January 1, 2016, to January 1, 2022. The criteria for the inclusion of patients were: the existence of an acutely occurring critical illness (patients with acute dysfunction of one or more organs or organ systems or a threatening risk for its occurrence, with the need for intensive follow-up and monitoring) and a stay in the ICU for longer than 48 h. Patients were excluded from the study if they were younger than 18 years, on a chronic hemodialysis program, with decompensated liver cirrhosis, with chronic gastrointestinal diseases (gastroesophageal reflux disease, peptic ulcer, gastritis, inflammatory bowel disease, irritable colon, chronic diarrhea, etc.), if the data from the medical history were not complete, and if a patient was a pregnant woman. The sample used for the study was of convenient type, although consecutive, i.e., all patients who satisfied inclusion and without exclusion criteria within the study period were included.

The main study outcomes were GID in general, and its specific types: paralytic ileus, gastroesophageal reflux, nausea, vomiting, and constipation. The specific types of GID were accounted for each of the study patients only if their diagnoses were recorded in the patient files by the responsible physician during the patients’ stay in the ICU, using the ICD-10 codes: K56.0 for paralytic ileus, K21.0 for gastroesophageal reflux, R11.0 for nausea, R11.1, R11.2, R11.10, R11.11, R11.12, and K91.0 for vomiting, and K59.01 for constipation. The patient was considered to have GID in general if at least one of the abovementioned specific types of GID was recorded in his/her file. Apart from several independent variables known from previous research that may influence gastrointestinal motility (opioids, previous abdominal surgery, mechanical ventilation, sepsis, shock, and diabetes mellitus), a plethora of confounders was also extracted from the patient files: routine blood biochemistry and hematology, Charlson comorbidity index, prescribed medication, and diagnoses of acute events occurring during hospitalization in the ICU. The data were extracted by two independent investigators from electronic patients’ histories embedded in the hospital information system “ZIS” (Comtrade, Belgrade) and then harmonized among themselves by consensus.

A minimal sample size was sufficient for the purpose of finding factors significantly associated with GID as the main outcome was calculated by the Schlesselman’s method [11]. The following inputs were used for the calculation: probability of type 1 error of 0.05, minimal statistical power 0.8, incidence of the outcome 51%, prevalence of inhalation injury as a risk factor of 54%, and meaningful adjusted odds ratio of 1.61 for the risk factor. The minimal sample size satisfying the inputs was 68 patients per the study group, or in total 136 patients.

The data collected from the ZIS information system were first numerically coded, tabulated, and checked for errors by both investigators independently. The data were then described by measures of central tendency and variability (if continuous), or by frequencies and relative numbers and percentages (if categorical). Mean and standard deviation were used as descriptors of normally distributed continuous data, while median and interquartile range described the data distributed in other way. Effects of putative predictors and confounders on the study outcomes were analyzed by multivariate binary logistic regression. Before applying these multivariate techniques, their assumptions were checked whether being satisfied (binary outcome, independency of observations, no multicollinearity, no extreme outliers, and sufficiently large sample for multivariate binary logistic regression). The quality of the regression models was checked by the Hosmer and Lemeshow test, Cox & Snell R square, and Nagelkerke R square. The results were considered statistically significant if the probability of null hypothesis was 0.05 or below. All calculations were made by the Statistical Package for the Social Sciences (SPSS), version 18.0.


**Ethical approval:** The research has been complied with all the relevant national regulations, institutional policies, and in accordance the tenets of the Helsinki Declaration and has been approved by the institutional review board, the Ethics Committee of University Clinical Center Kragujevac (approval number: 01/22-198).
**Informed consent**: This is a retrospective study, and for this type of study, formal consent is not required.

## 3 Results

The study included a total of 185 patients treated in the ICU. The number of cases that had ileus is 95 (51.4%), and the total number of controls is 90 (48.6%). Patients were matched by gender and age. No controls were found for 5 subjects. A total of 121 patients (65.4%) had gastro-esophageal reflux. Nausea was experienced by 92 patients (49.7%). Vomiting was experienced by 60 patients (32.4%). Constipation was experienced by 104 patients (56.2%). A total of 160 patients (86.5%) had some form of GID. Detailed data of the study sample of patients are shown in Table 1.

**Table 1:** Characteristics of respondents

Parameter	Value
Gender (m/f)	103/82 (55.7%/44.3%)
Age	65.55 ± 16.128, 69, 19
Ileus (yes/no)	95/90 (51.4%/48.6%)
Gastro-esophageal reflux (yes/no)	121/64 (65.4%/34.6%)
Nausea (yes/no)	92/93 (49.7%/50.3%)
Vomiting (yes/no)	60/125 (32.4%/67.6%)
Constipation (yes/no)	104/81 (56.2%/43.8%)
Mechanical ventilation (yes/no)	138/47 (74.6%/25.4%)
Recent abdominal surgeries (yes/no)	51/134 (27.6%/72.4%)
Sepsis (yes/no)	86/99 (46.5%/53.5%)
Diabetes mellitus 2 (yes/no)	122/63 (65.9%/34.1%)
Shock (yes/no)	89/96 (48.1%/51.9%)
Myocardial infarction (yes/no)	51/134 (27.6%/72.4%)
Congestive heart failure (yes/no)	138/47 (74.6%/25.4%)
Peripheral vascular disease (yes/no)	66/119 (35.7%/64.3%)
Cerebrovascular event (yes/no)	62/123 (33.5%/66.5%)
Dementia (yes/no)	23/162 (12.4%/87.6%)
Chronic obstructive pulmonary disease (yes/no)	85/100 (45.9%/54.1%)
Connective tissue disease (yes/no)	3/182 (1.6%/98.4%)
Gastric ulcer (yes/no)	95/90 (51.4%/48.6%)
Liver disease (yes/no)	7/178 (3.8%/96.2%)
Hemiplegia (yes/no)	25/160 (13.5%/86.5%)
Solid tumor (yes/no)	104/81 (56.2%/43.8%)
Acute kidney injury (yes/no)	88/97 (47.6%/52.4%)
Fatality (yes/no)	91/94 (49.2%/50.8%)
Antidiabetics (yes/no)	116/69 (62.7%/37.3%)
Antihypertensives (yes/no)	121/64 (65.4%/34.6%)
Antibiotics (yes/no)	160/25 (86.5%/13.5%)
Opioids (yes/no)	112/73 (60.5%/39.5%)
NSAIDs (yes/no)	129/56 (69.7%/30.3%)
PPIs (yes/no)	135/50 (73.0%/27.0%)
Antidepressants (yes/no)	32/153 (17.3%/82.7%)
Antipsychotics (yes/no)	17/168 (9.2%/90.8%)
H2 blockers (yes/no)	55/130 (29.7%/70.3%)
Corticosteroids (yes/no)	31/154 (16.8%/83.2%)
Heparin (yes/no)	129/56 (69.7%/30.3%)
Pulse	94.92 ± 22.412, 94, 32
Respiration rates	21.74 ± 5.705, 19.5, 10
Platelets	226.41 ± 146.354, 202, 194
Serum creatinine	151.2 ± 120.251, 110, 133
Sodium	140.423 ± 8.3323, 139, 7
Potassium	4.16 ± 0.891, 4.1, 1.0
Hematocrit	0.3109 ± 0.05992, 0.30, 0.07
Leukocytes	14.675 ± 9.1162, 12.5, 8.3
QSOFA	1.57 ± 1.343, 2.0, 3
Glasgow Coma Scale	12.91 ± 2.395, 14.0, 3
Charlson comorbidity index	8.01 ± 3.767, 8, 5

Abbreviations: NSAIDs – non-steroidal anti-inflammatory drugs; PPIs – proton pump inhibitors; QSOFA – Quick Sequential Organ Failure Assessment score.

Note: Results for continuous variables are shown as mean ± standard deviation, median, interquartile range, and for categorical variables as frequency and percentages.

The characteristics of the subjects according to the groups formed based on the outcome (ileus, gastro-esophageal reflux, nausea, vomiting, constipation) are shown in Table 2. Since there was no normal distribution, the results for continuous variables are shown as median and interquartile range and for categorical variables as frequency and percentages. The results of the tests for examining the significance of the difference between groups, and for individual parameters, are shown in the last column of Table 2.

**Table 2:** Univariate analysis according to outcomes

Parameter	Gastrointestinal dysmotility	No gastrointestinal dysmotility	Probability of null hypothesis (*p*)
**Ileus**			
Gender (m/f)	48/47 (50.5%/49.5%)	55/35 (61.1%/38.9)	0.193
Age	70 (19)	68 (19)	0.094
Platelets	216 (198)	187 (188)	0.284
Serum creatinine	133 (135)	92 (119)	0.045
Pulse	97 (31)	90 (33)	0.185
Sodium	139 (7)	140 (9)	0.152
Potassium	4 (1.2)	4.2 (1)	0.070
Hematocrit	0.31 (0.07)	0.29 (0.07)	0.051
Leukocytes	12.2 (8.9)	12.8 (7.7)	0.979
QSOFA	1 (3)	2 (3)	0.747
Respiration rates	18 (10)	23 (10)	0.261
Charlson comorbidity index	8 (5)	8 (5)	0.069
Glasgow Coma Scale	14 (3)	13 (4)	0.984
Mechanical ventilation (yes/no)	85/10 (89.5%/10.5%)	53/37 (58.9%/41.1%)	0.000
Opioids (yes/no)	74/21 (77.9%/22.1%)	38/52 (42.2%/57.8%)	0.000
Recent abdominal surgeries (yes/no)	45/50 (47.4%/52.6%)	6/84 (6.7%/93.3%)	0.000
Sepsis (yes/no)	52/43 (54.7%/45.3%)	34/56 (37.8%/62.2%)	0.030
Diabetes mellitus 2 (yes/no)	69/26 (72.6%/27.4%)	53/37 (58.9%/41.1%)	0.069
Shock (yes/no)	53/42 (55.8%/44.2%)	36/54 (40.0%/60.0%)	0.045
Acute kidney injury (yes/no)	48/47 (50.5%/49.5%)	40/50 (44.4%/55.6%)	0.496
Operated (yes/no)	93/2 (97.9%/2.1%)	90/0 (100%/0%)	0.501
Antidiabetics (yes/no)	69/26 (72.6%/27.4%)	47/43 (52.2%/47.8%)	0.007
Antihypertensives (yes/no)	64/31 (67.4%/32.6%)	57/33 (63.3%/36.7%)	0.673
Antibiotics (yes/no)	79/16 (83.2%/16.8%)	81/9 (90.0%/10.0%)	0.252
Opioids (yes/no)	74/21 (77.9%/22.1%)	38/52 (42.2%/57.8%)	0.000
NSAIDs (yes/no)	62/33 (65.3%/34.7%)	67/23 (74.4%/25.6%)	0.231
PPIs (yes/no)	72/23 (75.8%/24.2%)	63/27 (70.0%/30.0%)	0.471
Antidepressants (yes/no)	25/70 (26.3%/73.7%)	7/83 (7.8%/92.2%)	0.002
Antipsychotics (yes/no)	13/82 (13.7%/86.3%)	4/86(4.4%/95.6%)	0.055
H2 blockers (yes/no)	27/68 (28.4%/71.6%)	28/62 (31.1%/68.9%)	0.811
Corticosteroids (yes/no)	11/84 (11.6%/88.4%)	20/70 (22.2%/77.8%)	0.082
Heparin (yes/no)	73/22 (76.8%/23.2%)	56/34 (62.2%/37.8%)	0.045
**Gastro-esophageal reflux**			
Gender (m/f)	66/55 (54.5%/45.5%)	37/27(57.8%/42.2%)	0.787
Age	69 (17)	66 (24)	0.312
Platelets	195 (188)	220 (223)	0.364
Serum creatinine	118 (136)	95 (113)	0.056
Pulse	94 (35)	90 (30)	0.714
Sodium	140 (8)	139 (7)	0.632
Potassium	4.1 (0.9)	3.8 (1.2)	0.072
Hematocrit	0.30 (0.07)	0.31 (0.1)	0.776
Leukocytes	12.7 (8.4)	12.3 (8.6)	0.691
QSOFA	2 (3)	1 (3)	0.290
Respiration rates	23 (10)	18 (9)	0.052
Charlson comorbidity index	9 (4)	6 (7)	0.000
Glasgow Coma Scale	13 (4)	14 (3)	0.258
Mechanical ventilation (yes/no)	92/29 (76.0%/24.0%)	46/18 (71.9%/28.1%)	0.660
Opioids (yes/no)	78/43 (64.5%/35.5%)	34/30 (53.1%/46.9%)	0.179
Recent abdominal surgeries (yes/no)	36/85 (29.8%/70.2%)	15/49 (23.4%/76.6%)	0.458
Sepsis (yes/no)	57/64 (47.1%/52.9%)	29/35 (45.3%/54.7%)	0.938
Diabetes mellitus 2 (yes/no)	85/36 (70.2%/29.8%)	37/27 (57.8%/42.2%)	0.125
Shock (yes/no)	64/57 (52.9%/47.1%)	25/39 (39.1%/60.9%)	0.102
Acute kidney injury (yes/no)	61/60 (50.4%/49.6%)	27/37 (42.2%/57.8%)	0.362
Operated (yes/no)	120/1 (99.2%/0.8%)	63/1 (98.4%/1.6%)	1.000
Antidiabetics (yes/no)	81/40 (66.9%/33.1%)	35/29 (54.7%/45.3%)	0.139
Antihypertensives (yes/no)	84/37 (69.4%/30.6%)	37/27 (57.8%/42.2%)	0.157
Antibiotics (yes/no)	104/17 (86%/14%)	56/8 (87.5%/12.5%)	0.946
Opioids (yes/no)	78/43 (64.5%/35.5%)	34/30 (53.1%/46.9%)	0.179
NSAIDs (yes/no)	82/39 (67.8%/32.2%)	47/17 (73.4%/26.6%)	0.529
PPIs (yes/no)	89/32 (73.6%/26.4%)	46/18 (71.9%/28.1%)	0.944
Antidepressants (yes/no)	18/103 (14.9%/85.1%)	14/50 (21.9%/78.1%)	0.321
Antipsychotics (yes/no)	8/113 (6.6%/93.4%)	9/55 (14.1%/85.9%)	0.161
H2 blockers (yes/no)	31/90 (25.6%/74.4%)	24/40 (37.5%/62.5%)	0.130
Corticosteroids (yes/no)	22/99 (18.2%/81.8%)	9/55 (14.1%/85.9%)	0.612
Heparin (yes/no)	84/37 (69.4%/30.6%)	45/19 (70.3%/29.7%)	1.000
**Nausea**			
Gender (m/f)	51/41 (55.4%/44.6%)	52/41 (55.9%/44.1%)	1.000
Age	70 (21)	68 (16)	0.297
Platelets	197 (207)	213 (182)	0.824
Serum creatinine	133 (135)	92(116)	0.017
Pulse	98 (33)	90 (35)	0.212
Sodium	139 (7)	140 (9)	0.085
Potassium	4.0 (1.3)	4.2 (0.9)	0.150
Hematocrit	0.31 (0.08)	0.30 (0.07)	0.839
Leukocytes	12.2 (8.6)	12.8 (7.9)	0.683
QSOFA	2 (3)	2 (3)	0.542
Respiration rates	19 (10)	23 (10)	0.147
Charlson comorbidity index	8 (5)	8 (5)	0.120
Glasgow Coma Scale	14 (4)	14 (3)	0.352
Mechanical ventilation (yes/no)	81/11 (88.0%/12.0%)	57/36 (61.3%/38.7%)	0.000
Opioids (yes/no)	70/22 (76.1%/23.9%)	42/51 (45.2%/54.8%)	0.000
Recent abdominal surgeries (yes/no)	41/51 (44.6%/55.4%)	10/83 (10.8%/89.2%)	0.000
Sepsis (yes/no)	51/41 (55.4%/44.6%)	35/58 (37.6%/62.4%)	0.023
Diabetes mellitus 2 (yes/no)	65/27 (70.7%/29.3%)	57/36 (61.3%/38.7%)	0.235
Shock (yes/no)	54/38 (58.7%/41.3%)	35/58 (37.6%/62.4%)	0.007
Acute kidney injury (yes/no)	48/44 (52.2%/47.8%)	40/53 (43%/57%)	0.271
Operated (yes/no)	90/2 (97.8%/2.2%)	93/0 (100%/0%)	0.472
Antidiabetics (yes/no)	65/27 (70.7%/29.3%)	51/42 (54.8%/45.2%)	0.038
Antihypertensives (yes/no)	62/30 (67.4%/32.6%)	59/34 (63.4%/36.6%)	0.682
Antibiotics (yes/no)	76/16 (82.6%/17.4%)	84/9 (90.3%/9.7%)	0.187
Opioids (yes/no)	70/22 (76.1%/23.9%)	42/51 (45.2%/54.8%)	0.000
NSAIDs (yes/no)	58/34 (63.0%/37.0%)	71/22 (76.3%/23.7%)	0.070
PPIs (yes/no)	70/22 (76.1%/23.9%)	65/28 (69.9%/30.1%)	0.434
Antidepressants (yes/no)	25/67 (27.2%/72.8%)	7/86 (7.5%/92.5%)	0.001
Antipsychotics (yes/no)	13/79 (14.1%/85.9%)	4/89 (4.3%/95.7%)	0.039
H2 blockers (yes/no)	27/65 (29.3%/70.7%)	28/65 (30.1%/69.9%)	1.000
Corticosteroids (yes/no)	13/79 (14.1%/85.9%)	18/75 (19.4%/80.6%)	0.451
Heparin (yes/no)	73/19 (79.3%/20.7%)	56/37 (60.2%/39.8%)	0.008
**Vomiting**			
Gender (m/f)	28/32 (46.7%/53.3%)	75/50 (60.0%/40.0%)	0.121
Age	68 (21)	69 (18)	0.992
Platelets	194 (197)	205 (204)	0.642
Serum creatinine	133 (140)	101 (121)	0.069
Pulse	101 (28)	90 (35)	0.014
Sodium	139 (8)	139 (7)	0.592
Potassium	4.0 (1.1)	4.1 (1)	0.192
Hematocrit	0.31 (0.07)	0.30 (0.07)	0.837
Leukocytes	12.2 (9.1)	12.7 (7.9)	0.932
QSOFA	2 (3)	1 (3)	0.891
Respiration rates	22 (11)	19 (10)	0.766
Charlson comorbidity index	7 (6)	8 (5)	0.150
Glasgow Coma Scale	13 (3)	14 (4)	0.951
Mechanical ventilation (yes/no)	51/9 (85.0%/15.0%)	87/38 (69.6%/30.4%)	0.038
Opioids (yes/no)	48/12 (80.0%/20.0%)	64/61 (51.2%/48.8%)	0.000
Recent abdominal surgeries (yes/no)	29/31 (48.3%/51.7%)	22/103 (17.6%/82.4%)	0.000
Sepsis (yes/no)	33/27 (55.0%/45.0%)	53/72 (42.4%/57.6%)	0.147
Diabetes Mellitus 2 (yes/no)	42/18 (70.0%/30.0%)	80/45 (64.0%/36.0%)	0.522
Shock (yes/no)	36/24 (60.0%/40.0%)	53/72 (42.4%/57.6%)	0.037
Acute kidney injury (yes/no)	34/26 (56.7%/43.3%)	54/71 (43.2%/56.8%)	0.119
Operated (yes/no)	59/1 (98.3%/1.7%)	124/1 (99.2%/0.8%)	1.000
Antidiabetics (yes/no)	42/18 (70.0%/30.0%)	74/51 (59.2%/40.8%)	0.208
Antihypertensives (yes/no)	40/20 (66.7%/33.3%)	81/44 (64.8%/35.2%)	0.932
Antibiotics (yes/no)	46/14 (76.7%/23.3%)	114/11 (91.2%/8.8%)	0.013
Opioids (yes/no)	48/12 (80.0%/20.0%)	64/61 (51.2%/48.8%)	0.000
NSAIDs (yes/no)	36/24 (60.0%/40.0%)	93/32 (74.4%/25.6%)	0.068
PPIs (yes/no)	48/12 (80.0%/20.0%)	87/38 (69.6%/30.4%)	0.189
Antidepressants (yes/no)	16/44 (26.7%/73.3%)	16/109 (12.8%/87.2%)	0.033
Antipsychotics (yes/no)	10/50 (16.7%/83.3%)	7/118 (5.6%/94.4%)	0.030
H2 blockers (yes/no)	13/47 (21.7%/78.3%)	42/83 (33.6%/66.4%)	0.136
Corticosteroids (yes/no)	11/49 (18.3%/81.7%)	20/105 (16.0%/84.0%)	0.851
Heparin (yes/no)	43/17 (71.7%/28.3%)	86/39 (68.8%/31.2%)	0.821
**Constipation**			
Gender (m/f)	56/48 (53.8%/46.2%)	47/34 (58.0%/42.0%)	0.676
Age	70 (18)	67 (21)	0.004
Platelets	213 (205)	187 (181)	0.452
Serum creatinine	142 (145)	89 (92)	0.000
Pulse	96 (32)	90 (34)	0.656
Sodium	139 (8)	139 (7)	0.790
Potassium	4.0 (1.3)	4.1 (0.9)	0.509
Hematocrit	0.31 (0.08)	0.3 (0.06)	0.392
Leukocytes	12.4 (9.8)	12.6 (7.3)	0.298
QSOFA	2 (3)	1 (3)	0.522
Respiration rates	22 (10)	19 (10)	0.329
Charlson comorbidity index	9 (6)	7 (6)	0.011
Glasgow Coma Scale	13 (4)	14 (3)	0.240
Mechanical ventilation (yes/no)	85/19 (81.7%/18.3%)	53/28 (65.4%/34.6%)	0.018
Opioids (yes/no)	77/27 (74.0%/26.0%)	35/46 (43.2%/56.8%)	0.000
Recent abdominal surgeries (yes/no)	42/62 (40.4%/59.6%)	9/72 (11.1%/88.9%)	0.000
Sepsis (yes/no)	55/49 (52.9%/47.1%)	31/50 (38.3%/61.7%)	0.067
Diabetes mellitus 2 (yes/no)	76/28 (73.1%/26.9%)	46/35 (56.8%/43.2%)	0.031
Shock (yes/no)	60/44 (57.7%/42.3%)	29/52 (35.8%/64.2%)	0.005
Acute kidney injury (yes/no)	59/45 (56.7%/43.3%)	29/52 (35.8%/64.2%)	0.007
Operated (yes/no)	103/1 (99.0%/1.0%)	80/1 (98.8%/1.2%)	1.000
Antidiabetics (yes/no)	73/31 (70.2%/29.8%)	43/38 (53.1%/46.9%)	0.026
Antihypertensives (yes/no)	69/35 (66.3%/33.7%)	52/29 (64.2%/35.8%)	0.882
Antibiotics (yes/no)	92/12 (88.5%/11.5%)	68/13 (84.0%/16.0%)	0.501
Opioids (yes/no)	77/27 (74.0%/26.0%)	35/46 (43.2%/56.8%)	0.000
NSAIDs (yes/no)	70/34 (67.3%/32.7%)	59/22 (72.8%/27.2%)	0.515
PPIs (yes/no)	79/25 (76.0%/24.0%)	56/25 (69.1%/30.9%)	0.384
Antidepressants (yes/no)	24/80 (23.1%/76.9%)	8/73 (9.9%/90.1%)	0.031
Antipsychotics (yes/no)	14/90 (13.5%/86.5%)	3/78 (3.7%/96.3%)	0.043
H2 blockers (yes/no)	28/76 (26.9%/73.1%)	27/54 (33.3%/66.7%)	0.433
Corticosteroids (yes/no)	14/90 (13.5%/86.5%)	17/64 (21.0%/79.0%)	0.245
Heparin (yes/no)	76/28 (73.1%/26.9%)	53/28 (65.4%/34.6%)	0.336

Abbreviations: NSAIDs – non-steroidal anti-inflammatory drugs; PPIs – proton pump inhibitors; QSOFA – Quick Sequential Organ Failure Assessment score.

Note: Results for continuous variables are shown as median and interquartile range, and for categorical variables as frequency and percentages.

Multivariate analysis was performed using binary logistic regression. The results are shown in Table 3, where crude odds ratio (OR) is the result of univariate logistic regression, and adjusted OR is the result of multivariate logistic regression.

**Table 3:** Multivariate analysis according to outcomes

Parameter	Crude OR	Adjusted OR	*p* (for adjusted OR)
**Ileus**			
Charlson comorbidity index	0.926 (0.855–1.002)	0.648 (0.531–0.792)	0.000
Acute kidney injury	1.277 (0.716–2.277)	4.737 (1.670–13.439)	0.003
Antidiabetics	2.428 (1.317–4.477)	2.969 (1.216–7.249)	0.017
Opioids	4.822 (2.542–9.146)	4.814 (2.038–11.368)	0.000
Antidepressants	4.235 (1.728–10.379)	4.225 (1.301–13.719)	0.016
Recent abdominal surgeries	12.600 (5.017–31.647)	14.143 (4.594–43.537)	0.000
Mechanical ventilation	5.934 (2.725–12.922)	4.060 (1.533–10.755)	0.005
Age	1.011 (0.992–1.029)	1.059 (1.023–1.097)	0.001
**Gastro-esophageal reflux**			
Charlson comorbidity index	1.176 (1.076–1.286)	1.266 (1.123–1.429)	0.000
Opioids	1.601 (0.864–2.964)	1.919 (0.993–3.706)	0.052
**Nausea**			
Charlson comorbidity index	0.938 (0.867–1.014)	0.854 (0.762–0.958)	0.007
Acute kidney injury	1.445 (0.810–2.580)	3.455 (1.448–8.243)	0.005
Opioids	3.864 (2.059–7.251)	3.216 (1.493–6.927)	0.003
Antidepressants	4.584 (1.870–11.241)	4.713 (1.683–13.200)	0.003
Heparin	2.539 (1.320–4.880)	3.075 (1.341–7.053)	0.008
Recent abdominal surgeries	6.673 (3.076–14.472)	5.894 (2.401–14.470)	0.000
Mechanical ventilation	4.651 (2.185–9.900)	3.398 (1.413–8.169)	0.006
**Vomiting**			
Charlson comorbidity index	0.939 (0.864–1.021)	0.864 (0.775–0.964)	0.009
Acute kidney injury	1.719 (0.924–3.200)	3.914 (1.731–8.847)	0.001
Opioids	3.812 (1.850–7.858)	3.491 (1.561–7.810)	0.002
Antidepressants	2.477 (1.140–5.384)	2.563 (1.077–6.098)	0.033
Recent abdominal surgeries	4.380 (2.209–8.683)	4.428 (2.082–9.414)	0.000
**Constipation**			
Acute kidney injury	2.351 (1.294–4.272)	3.112 (1.502–6.450)	0.002
Opioids	3.748 (2.015–6.973)	4.572 (2.187–9.558)	0.000
Antipsychotics	4.044 (1.121–14.594)	3.187 (0.819–12.405)	0.095
Recent abdominal surgeries	5.419 (2.445–12.013)	5.288 (2.168–12.896)	0.000
Age	1.032 (1.011–1.052)	1.033 (1.010–1.057)	0.005

Abbreviation: OR – odds ratio.

Association of independent and confounding variables with ileus was tested by multivariate binary logistic regression. The model was built by backward conditional stepwise method starting with a full set of potential predictors: serum creatinine, acute kidney injury, Charlson comorbidity index, prescribed antidiabetics, opioids, antidepressants, antipsychotics, heparin, sepsis, recent abdominal surgery, and mechanical ventilation. The assumptions of binary outcome (ileus or not), independency of observations, no multicollinearity (variance inflation factor – VIF was below 2 for all predictors), no extreme outliers, and sufficiently large sample were all met. The linear relationship between explanatory variables and the logit of the outcome was confirmed for all variables by the Box-Tidwell test. The final model of binary logistic regression included variables shown in Table 3 and was a satisfactory fit of the data: Hosmer and Lemeshow test was 14.793 (df = 8, *p* = 0.063), Cox & Snell *R* square 0.423, and Nagelkerke *R* square 0.564.

The association of independent and confounding variables with gastro-esophageal reflux was tested by multivariate binary logistic regression. The model was built by backward conditional stepwise method starting with a full set of potential predictors: serum creatinine, acute kidney injury, Charlson comorbidity index, prescribed antidiabetics, opioids, antidepressants, antipsychotics, heparin, sepsis, recent abdominal surgery, and mechanical ventilation. The assumptions of binary outcome (gastro-esophageal reflux or not), independency of observations, no multicollinearity (variance inflation factor – VIF was below 2 for all predictors), no extreme outliers, and sufficiently large sample were all met. The linear relationship between explanatory variables and the logit of the outcome was confirmed for all variables by the Box-Tidwell test. The final model of binary logistic regression included variables shown in Table 3 and was a satisfactory fit of the data: Hosmer and Lemeshow test was 4.164 (df = 8, *p* = 0.842), Cox & Snell *R* square 0.118, and Nagelkerke *R* square 0.163.

The association of independent and confounding variables with nausea was tested by multivariate binary logistic regression. The model was built by backward conditional stepwise method starting with a full set of potential predictors: serum creatinine, acute kidney injury, Charlson comorbidity index, prescribed antidiabetics, opioids, antidepressants, antipsychotics, heparin, sepsis, recent abdominal surgery, and mechanical ventilation. The assumptions of binary outcome (nausea or not), independency of observations, no multicollinearity (variance inflation factor – VIF was below 2 for all predictors), no extreme outliers, and sufficiently large sample were all met. The linear relationship between explanatory variables and the logit of the outcome was confirmed for all variables by the Box-Tidwell test. The final model of binary logistic regression included variables shown in Table 3 and was a satisfactory fit of the data: Hosmer and Lemeshow test was 11.986 (df = 8, *p* = 0.152), Cox & Snell *R* square 0.362, and Nagelkerke *R* square 0.482.

The association of independent and confounding variables with vomiting was tested by multivariate binary logistic regression. The model was built by backward conditional stepwise method starting with a full set of potential predictors: serum creatinine, acute kidney injury, Charlson comorbidity index, prescribed antidiabetics, opioids, antidepressants, antipsychotics, heparin, sepsis, recent abdominal surgery, and mechanical ventilation. The assumptions of binary outcome (vomiting or not), independency of observations, no multicollinearity (variance inflation factor – VIF was below 2 for all predictors), no extreme outliers, and sufficiently large sample were all met. The linear relationship between explanatory variables and the logit of the outcome was confirmed for all variables by the Box-Tidwell test. The final model of binary logistic regression included variables shown in Table 3 and was a satisfactory fit of the data: Hosmer and Lemeshow test was 8.460 (df = 8, *p* = 0.390), Cox & Snell *R* square 0.228, and Nagelkerke *R* square 0.319.

The association of independent and confounding variables with constipation was tested by multivariate binary logistic regression. The model was built by backward conditional stepwise method starting with a full set of potential predictors: serum creatinine, acute kidney injury, Charlson comorbidity index, prescribed antidiabetics, opioids, antidepressants, antipsychotics, heparin, sepsis, recent abdominal surgery, and mechanical ventilation. The assumptions of binary outcome (constipation or not), independency of observations, no multicollinearity (variance inflation factor – VIF was below 2 for all predictors), no extreme outliers, and sufficiently large sample were all met. The linear relationship between explanatory variables and the logit of the outcome was confirmed for all variables by the Box-Tidwell test. The final model of binary logistic regression included variables shown in Table 3 and was a satisfactory fit of the data: Hosmer and Lemeshow test was 6.309 (df = 8, *p* = 0.613), Cox & Snell *R* square 0.277, and Nagelkerke *R* square 0.371.

## 4 Discussion

Significant risk factors associated with some form of GID in our study were acute kidney injury (associated with ileus, nausea, vomiting, and constipation), recent abdominal surgery (associated with ileus, nausea, vomiting, and constipation), mechanical ventilation (associated with ileus and nausea), advanced age (associated with ileus and constipation), as well as the use of certain drugs such as opioids (associated with ileus, nausea, vomiting, and constipation), antidepressants (associated with ileus, nausea, and vomiting), and antidiabetics (associated with ileus only). However, Charlson comorbidity index had a divergent effect depending on the form of GID: it increased the risk of gastroesophageal reflux, but it was protective against ileus, nausea, and vomiting.

Previous studies have found that comorbidities are very common in patients with gastro-esophageal reflux [12], what could explain our finding that Charlson comorbidity index is associated with an increased risk of gastro-esophageal reflux. In a study on patients with colon cancer that were subjected to surgical resection, the age-adjusted Charlson comorbidity index was an independent predictor of the development of extended postoperative ileus [13]. In contrast, we found that Charlson comorbidity index was protective against ileus, as well as against nausea and vomiting. Given that our study was conducted in the ICU, it is possible that physicians were dedicated more to patients with high comorbidity burden, and therefore more often applied some prophylactic measures against ileus, nausea, or vomiting in these patients. A significant risk factor for the development of ileus in our study was acute kidney injury. As part of the acute kidney injury, electrolyte and hormone imbalance occur, together with metabolic abnormalities [14], which may cause ileus, often with bloating, vomiting or hiccup [15]. Development of ileus was favored by antidiabetic drugs, especially by metformin. Since the patients on therapy with metformin often have lactic acidosis, it could explain the emergence of paralysis of small and large bowel [16]. The well-known inhibitory effect of opioids on motility of small and large bowel has been confirmed in our study; this effect is mediated by the activation of peripheral μ-opioid receptors [17]. The association of administration of antidepressants with the appearance of ileus in our study was also not surprising. Particularly, tricyclic and many heterocyclic antidepressants have an anti-cholinergic effect, i.e., directly block muscarine M3 receptors involved in the initiation and propagation of normal peristaltic waves [18].

A recent surgery in the abdomen is another factor associated with the appearance of ileus; however, the mechanism of its inhibitory effect is unknown. Due to the activation of pain receptors, abdominal surgery leads to the hyperactivity of the sympathetic nervous system and hypertonia of intestinal sphincters; besides, electrolyte disorders that accompany blood loss, and an intensive administration of crystalloid solutions during and after operations can inhibit depolarization of neurons in the myenteric plexus and prevent initiation of peristalsis [19]. Mechanical ventilation, which is often administered to surgical patients, further leads to the inhibition of peristalsis, because it reduces blood flow through abdomen, so the resulting hypoxia inhibits the functioning of myenteric neurons and smooth muscle cells [20]. As mechanical ventilation, antidiabetic drugs and opioids are more often used in elderly patients, who otherwise have a slow passage of intestinal contents, it is not surprising that the age in our study was associated with ileus [21].

Over 50% of our patients had nausea, so a large number of factors were associated with its occurrence. First, patients with acute kidney injury had nausea due to the acidosis and accumulation of degradational metabolic products [14,22]. The administration of opioids also provoked nausea, which is known to be mediated through the μ-receptors in the chemoreceptor zone, especially in patients who have never taken opioids earlier (this was the most often the case with acutely ill critical patients) [23]. Nausea was more often present in our patients on chronic therapy with selective serotonin reuptake inhibitors. These drugs, and among them, especially fluoxetine, cause nausea as an unwanted effect due to interference with serotonergic transmission in the stomach wall, especially in the mucosal layer [24]. All factors associated with nausea also made patients prone to vomiting [25,26].

Postoperative vomiting was frequent in our patients; those submitted to longer operations experienced more often vomiting after the surgery: prolongation of surgery for 30 min increased the risk of vomiting by about 60% [27]. At least part of this phenomenon could be explained by effects of nitrogen-suboxide (N_2_O), which increases pressure in semicircular channels of internal ear, and also in the abdomen due to its passage through esophagus and stomachs into the small bowel during general anesthesia [27,28].

Constipation occurred in our study more often in patients with acute kidney injury. Possible reasons for the development of constipation in these patients are accumulation of inflammatory mediators and degradational products of metabolism in the GIT [29] and modification of the intestinal flora (dysbiosis) due to inadequate water intake and secretion of urea from the intestinal wall [30]. This hypothesis is further supported by the results of clinical studies where probiotics or fecal transplants helped establishing not only normal intestinal flora but also normal motility [31]. The administration of opioids also contributed to the development of constipation due to the stimulation of opioid receptors in the wall of intestines, which further inhibited myenteric neurons and smooth muscle cells [32]. Finally, patients after abdominal surgery often have constipation, which can be explained by low food intake, immobility, and depression that are often encountered postoperatively [33]. Elderly are significantly more susceptible to constipation due to reduced fluid intake, slowing of intestinal transit, and frequent polypharmacy [34,35].

This study has several limitations. As a unicentric study, it is subject to effect of local factors on outcomes (e.g., local treatment protocols, unstandardized medical practice, unavailability of certain drugs). A relatively small number of patients and the small statistical power of this study have created conditions for falsely negative results, e.g., effect of antipsychotics did not reach significance because they were prescribed to a small percentage of patients. Due to the unavailability of some laboratory analyses during the study (bilirubin, partial pressure of oxygen, fraction of oxygen in inspired air, and blood pH), we could not calculate Apache II score for many patients.

## 5 Conclusions

In conclusion, the factors we identified as associated with the GID should be kept in mind by physicians who work with critically ill patients in an ICU. Early detection of these factors enables the implementation of measures that can either prevent, or at least mitigate the GID, and thus accelerate the recovery of critically ill.
